# Reduced Neutrophil Extracellular Trap Formation During Ischemia Reperfusion Injury in C3 KO Mice: C3 Requirement for NETs Release

**DOI:** 10.3389/fimmu.2022.781273

**Published:** 2022-02-16

**Authors:** Xiaoting Wu, Danyu You, Jiong Cui, Liyan Yang, Liyu Lin, Yi Chen, Changsheng Xu, Guili Lian, Jianxin Wan

**Affiliations:** ^1^ Department of Nephrology, Blood Purification Research Center, the First Affiliated Hospital of Fujian Medical University, Fuzhou, China; ^2^ Fujian Hypertension Research Institute, the First Affiliated Hospital of Fujian Medical University, Fuzhou, China

**Keywords:** ischemia reperfusion injury, acute kidney injury, complement component 3, neutrophils, neutrophil extracellular traps, peptidylarginine deiminase 4

## Abstract

Complement C3 plays a prominent role in inflammatory processes, and its increase exacerbates ischemia reperfusion injury (IRI)-induced acute kidney injury (AKI). Infiltrated neutrophils can be stimulated to form neutrophil extracellular traps (NETs), leading to renal injury. However, the relationship between the increase of C3 and the release of NETs in AKI was not clear. Here we found that IRI in the mouse kidney leads to increased neutrophils infiltration and NET formation. Furthermore, neutrophils depletion by anti-Ly6G IgG (1A8) did not reduce C3 activation but reduced kidney injury and inflammation, indicating a link between neutrophils infiltration and renal tissue damage. Pretreatment with 1A8 suppressed ischemia-induced NET formation, proving that extracellular traps (ETs) in renal tissue were mainly derived from neutrophils. Renal ischemia injury also leads to increased expression of C3. Moreover, C3 KO mice (C3 KO) with IRI exhibited attenuated kidney damage and decreased neutrophils and NETs. *In vitro*, C3a primed neutrophils to form NETs, reflected by amorphous extracellular DNA structures that colocalized with CitH3 and MPO. These data reveal that C3 deficiency can ameliorate AKI by reducing the infiltration of neutrophils and the formation of NETs. Targeting C3 activation may be a new therapeutic strategy for alleviating the necroinflammation of NETs in AKI.

## Introduction

Ischemia–reperfusion injury (IRI) is a major cause of acute kidney injury (AKI), which correlates with high morbidity, mortality, and seriously threatens human health (1). The pathophysiology of IRI consists of reactive oxygen species, neutrophil activation, and complement cascade activation (2).

Neutrophils are typically the first inflammatory cells to infiltrate the renal interstitium after AKI onset (3). Excessive neutrophils accumulation can induce tissue injury. In addition to exhibiting phagocytosis, neutrophils have been confirmed to release neutrophil extracellular traps (NETs). Peptidylarginine deiminase 4 (PAD4) is required for generation of NETs, by promoting histone citrullination and release of decondensed chromatin and granule content (4, 5). Interestingly, recent research has reported that ETs can also be released by cells other than neutrophils (6), such as macrophages (7, 8), eosinophils (9), and mast cells (10, 11). NETs were initially described as a part of the antimicrobial defense and were recently demonstrated to be involved in the pathogenesis of sterile inflammation, such as renal IRI (12). Neutrophil proteins such as MPO or Ly6g co-expression with citrullinated histone H3 (CitH3) is considered as evidence of NET formation (13).

The complement system plays a prominent role in host defense; nevertheless, it can cause significant tissue injury if not properly controlled (14, 15). The kidney seems vulnerable to complement attack and is involved in the pathogenesis of various kidney diseases as an inflammatory factor. As the convergence point of the complement system, C3 is indispensable for all three pathways of complement activation (16). C3aR is the receptor of C3 and plays a vital role in the pathophysiological function of C3. Following ischemia–reperfusion, C3 likely has increased access to the interstitial space. These lead to inflammation, promotion of extrinsic and intrinsic mononuclear cells (macrophages and dendritic cells), and direct cellular injury (15).

The interaction between complement and NETs has received increasing attention and has been reported in sepsis (17), ANCA-associated vasculitides (18), and small bowel tumors (19). Although both neutrophils and C3 make essential contributions to the pathogenesis of AKI, the interaction between them has not been reported.

We hypothesized that C3 increase after IRI can promote neutrophils to release NETs, which further plays an important role during ischemia-reperfusion induced renal injury, and that C3 KO may inhibit NETs release and protect renal function. To verify this hypothesis, we clamped the bilateral renal pedicles to induce ischemia–reperfusion injury in mice and analyzed the expression of NETs and C3. We injected Ly6G-specific monoclonal antibody intraperitoneally to deplete neutrophils *in vivo*. To further validate that C3 can promote the release of NETs, we compared renal injury between WT mice and C3 KO mice after IRI.

## Methods

### Acute Ischemia/Reperfusion Injury in Mice

All animal protocols were reviewed and approved by the Institutional Animal Care and Use Committee of Fujian Medical University (Approval Number: 2017-062). All animal experiments were carried out with randomized methods. Adult male wild-type (WT) C57BL/6J mice 8 to 12 weeks old were obtained from the Shanghai Laboratory Animal Center (Shanghai, China), C3 KO (strain B6.129S4-C3^tm1Crr^) mice (C57BL/6J mice background) were purchased from the Jackson Laboratory (Bar Harbor, ME, USA). Mice were anesthetized by intraperitoneal injection of 2% pentobarbital sodium and kept in a warm surgical environment at 37°C. The abdomens of the mice was opened layer-by-layer, and both kidney pedicles were clamped with micro-arterial clips for 45 min. After 45 min, the clips were removed carefully, the kidney was confirmed for recovery of blood flow by returning from dark red to bright red. Mice were sacrificed after reperfusion 6, 12, 24, and 48 hours. Control mice did not undergo the surgical procedure. Sham group mice underwent the same surgical procedure without clamping the kidney pedicles. The blood was sampled by the method of cardiac puncture.

### Detection of Neutrophils Count, Serum Creatinine, and Urea

The whole blood from mice was collected into an EDTA tube and blood routine examination was performed in the Laboratory Department of our hospital. The blood obtained from mice was collected into an eppendorf tube and centrifuged at 500×*g* at 4°C for 10 min to separate serum. An autoanalyzer analyzed serum creatinine and urea at the Laboratory Department of our hospital.

### Depletion of Neutrophils

For neutrophils depletion, we intraperitoneally injected 500 ug rat anti-mouse Ly6G antibody (1A8; BioXCell) 24 and 2 h before IRI surgery. The control group received the same dose of control IgG (IgG) at the same time. Differential leukocyte counts detected depletion of neutrophils in the blood.

### Degradation of NETs

The formation of NETs was prevented *in vivo* by intraperitoneally injected PAD4I specific inhibitor GSK 484 (MCE, USA, HY-100514) (10 mg/kg in 200 ul of 2% DMSO in NaCl) or vehicle (200 ul of 2% DMSO in NaCl), twice daily from −1 to day +1 (IRI model).

### Histology and Immunohistochemistry

After reperfusion, mice were euthanized at 6, 12, 24, and 48 h. Kidneys were preserved in 4% paraformaldehyde and embedded in paraffin. Kidney sections (4 μm) were dewaxed and rehydrated. Sections were blocked with 5% bovine serum albumin for 30 min at 37°C. The sections were incubated with specific antibodies against C3 (1:500, Santa Cruz Biotechnology, Cat# sc-28294, RRID: AB_627277), ICAM-1 (1:1,000 Servicebio, Cat#GB11106), MPO (1:1,000 Servicebio, Cat# GB11224), and Ly6G (1:400 Servicebio, Cat# GB11229) for 1 h followed by the appropriate horseradish peroxidase-conjugated secondary antibodies. Tubular necrosis in HE staining was scored by assessing the percentage of tubules in the corticomedullary junction that displayed tubular cell necrosis, tubular dilation, cast formation, and inflammatory cell infiltration.

### Immunofluorescent Staining

Half renal tissue sections were fixed in paraformaldehyde for 20 min and incubated in 2% FBS for 30 min to avoid nonspecific binding. Then, the sections were incubated with specific primary antibodies against citrullinated-histone H3 (CitH3 1:200; Abcam, Cat# ab5103, RRID: AB_304752), Ly6G (1:100; Santa Cruz Biotechnology, Cat# sc-53515, RRID: AB_783639), overnight at 4°C. Sections were washed with PBS and incubated with Alexa Fluor 488 or Alexa Fluor 594 conjugated goat anti-rabbit or goat anti-rat IgG for 1 h at 37°C. Sections were incubated with 4’,6-diamidino-2-phenylindole DAPI for nuclear staining. The images were visualized using a fluorescence microscope. NETs were defined as having a widespread network-like extracellular structure.

### Terminal Deoxynucleotidyl Transferase dUTP Nick End Labeling (TUNEL) Fluorescent Staining

Mice renal paraffin slices were dewaxed and hydrated. The sections were then permeabilized with 20 ug/ml of DNA-free Proteinase K (20 min, room temperature). The reaction mixture, including the TdT enzyme, was added and incubated continuously in the dark for 60 min at 37°C. Then, they were washed 3 times with 1× PBS and restained with DAPI. Fluorescence microscopy was used to examine the slices obtained.

### Western Blot

Renal tissues were lysed in ice-cold RIPA buffer containing phenylmethanesulfonyl fluoride (PMSF) for 30 min at 4°C. Protein concentrations were quantified using a BCA protein assay kit. The protein samples were separated by 6–15% SDS-PAGE and transferred to PVDF membrane, followed by blocking with 5% skim milk for 1 h. After that, membranes were incubated overnight using the following antibodies: CitH3 (1:1,000, Abcam, Cat# ab5103, RRID : AB_304752), C3aR (1:500, Santacruz, Cat# 133172, RRID : AB_2066736), PAD4 (1:1,000, Abcam, Cat# ab214810) and βactin (1:1,000, Santacruz, Cat# 47778, RRID : AB_2714189) as an internal control. The membranes were subsequently incubated with appropriate secondary antibodies for 1 h at room temperature. Specific bands were detected by ECL.

### Quantitative Real-Time RT-PCR

Total RNA was isolated from renal tissue using Trizol. cDNA was synthesized from total RNA using a cDNA synthesis kit (Roche). The SYBR Green Dye detection system was used for quantitative real-time PCR on a Light Cycler 480 (Roche). The reverse and forward gene-specific primers used in this study are listed as follows: mouse C3 (forward, 5’-CGGTGTGCTGAAGAGAACT-3’; reverse, 5’-ACTTGATGTGGCTCTGATGAACT-3’); mouse GAPDH (forward, 5’-TGGTGAAGGTCGGT-GTGAAC-3’; reverse, 5’-GAATTTGCCGTGAGTGGAGTC-3’). C3 gene expression was normalized using GAPDH as a housekeeping gene.

### ELISA

The frozen renal tissue was washed with cold PBS and weighed. One gram of the tissue was diluted per 9 ml of cold PBS with protease inhibitor and ground at freezing temperature to obtain homogenate. The lysate was centrifuged at 5,000×*g* for 15 min and the supernatant was collected. Protein levels of C3 in renal tissue were detected by mouse C3 ELISA kits (R&D system) according to the manufacturer’s instructions. The OD was measured by a multimode ELISA plate reader (Tecan, Sand Springs, OK, USA)

### Human Neutrophils Isolation and *In Vitro* NETs Experiments

We obtained 10 ml peripheral blood of healthy volunteers in EDTA-anticoagulated tubes. Neutrophils were isolated using Polymorphprep following the manufacturer’s protocol. The purity of neutrophils was performed using Wright-Giemsa staining. Neutrophils were seeded (2 × 10^5^ cells/ml) into 12 well plates in RPMI supplemented with 10% fetal bovine serum, and 1% penicillin–streptomycin allowed to adhere for 30 min and then stimulated with 15 nM PMA, RPMI, 0.01, 0.1, and 1 μM C3a in a 5% carbon dioxide atmosphere at 37°C for 4 h.

### Wright–Giemsa Staining

Morphological assessment of peripheral blood obtained from mice or healthy volunteers was performed using Wright–Giemsa (Wuhan Servicebio Technology, Wuhan, China) staining according to the manufacturer’s protocol on slides. The morphology of cells or blood was observed under a light microscope.

### Statistical Analyses

Data are expressed as the means ± SD. Normality of distribution has been tested using Shapiro–Wilk before analysis. Comparison between groups was performed by one-way ANOVA using Tukey test for *post hoc* assessments. A value of P < 0.05 was considered statistically significant. All statistical analyses were calculated with GraphPad Prism 7 software. *P* < 0.05 was considered statistically significant.

## Results

### Neutrophils Infiltration Increased After Renal IRI

**Figure 1:**
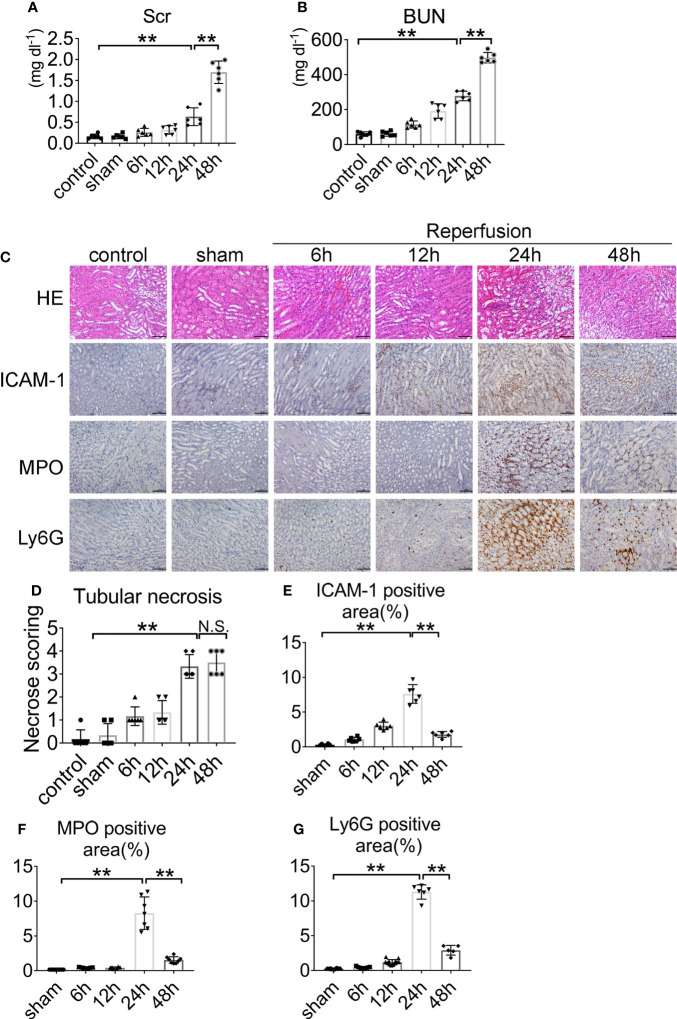
Renal function and representative histology of kidney after renal IRI. **(A)** Serum creatinine (Scr) and **(B)** blood urea nitrogen (BUN) levels of WT mice with clamping time of the renal artery of 45 min and reperfusion for 6, 12, 24, 48 h or sham surgery, n = 5–6/group. **(C)** Histology of IRI kidney at different time points after reperfusion. HE staining (Row 1), ICAM-1 immunohistochemistry (Row 2), MPO immunohistochemistry (Row 3) and Ly6G immunohistochemistry (Row 4). Scale bar = 200 μM. The percentage of positive neutrophil staining in 10 non-overlapping fields was quantified using Image J software. **(D)** Score for histopathology of tubular damage after renal IRI using HE stained renal tissue sections. **(E)** ICAM-1-positive area. **(F)** MPO-positive area. **(G)** Ly6G-positive area. n = 5–6/group. **P < 0.01. N.S., No Significance. Data are presented as mean ± SD.

Renal IRI was induced by clamping both renal arteries pedicle for 45 min. Mice were sacrificed after 6, 12, 24, and 48 h. Serum creatinine and serum urea increased threefold over baseline at 45 min after renal ischemia and 24 h after reperfusion (**Figures 1A, B**). Kidney sections from control and sham-operated mice displayed normal morphology, with well-preserved brush border membranes and no loss of tubular epithelial cells. Kidneys from wild type (WT) mice subjected to IRI showed necrotic effects on renal tissues, as reflected by loss of the brush border, loss of tubular epithelial cells, cast formation, and tubular dilation. There was extensive tubular epithelial cell necrosis and massive neutrophils infiltration in the kidney outer medulla 24 h after reperfusion (**Figure 1C**). The tubular injury score was used to grade renal tubular necrosis in hematoxylin & eosin (HE)-stained kidney sections from mice after IRI (**Figure 1D**). ICAM-1, MPO, and Ly6G proteins were measured in kidney tissue 6, 12, 24, and 48 h after reperfusion or sham surgery. At 24 h of renal reperfusion, the abundant presence of neutrophils was present in the corticomedullary region and greater than that in sham mice. The degree of cortical and medullary damage after IRI was inconsistent, with neutrophil infiltration concentrated at the corticomedullary junction, which suffered the greatest extent of the injury (**Figures 1C, E–G**). At the 6, 12, 24, and 48 h after reperfusion, the mice peripheral blood was observed by optical microscope *via* Wright–Gimsa staining (**Supplementary Figure S1A**). After the bilateral renal injury, the circulating neutrophils content was greatly increased, and the 24 h point was the peak (**Supplementary Figure S1B**). These data support the conclusion that renal IRI induces neutrophils upregulation and activity.

### NETs are Upregulated in Mouse Kidney After IRI

**Figure 2:**
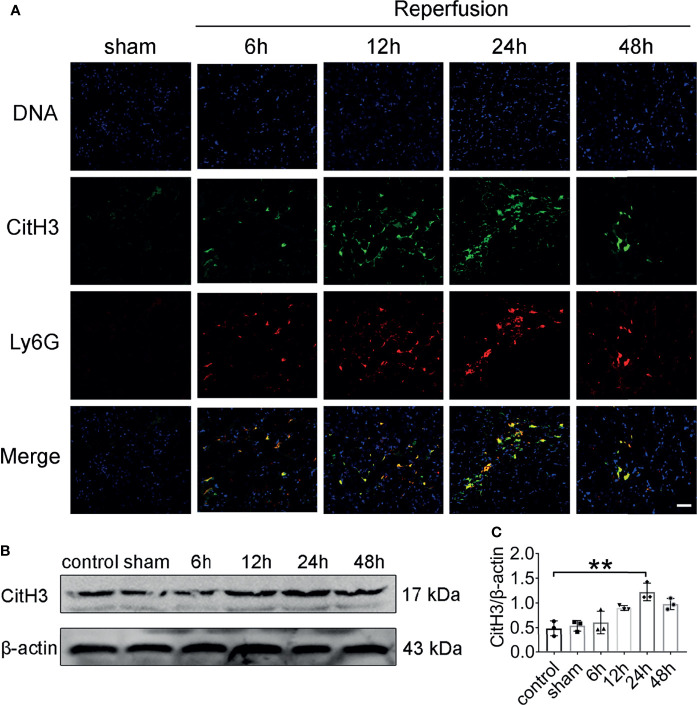
NET formation in the mouse kidney after acute injury. **(A)** Representative NETs staining of bilateral IRI at different time points after reperfusion. Colocalization of CitH3 (green), Ly6G (red), and swelled nuclei (blue). **(B)** CitH3 (expected weight: 17 kDa) expression in bilateral IRI kidneys by immunoblotting. **(C)** Quantitative analysis was analyzed by normalization to β-actin expression, n = 3/group. **P < 0.01. Data are presented as mean ± SD.

To investigate whether NETs are formed *in vivo* after renal IRI, we stained kidney sections for CitH3 and Ly6G. Intact neutrophils were observed in kidney sections of mice after sham surgery (**Figure 2A**). In addition, the formation of NETs was observed in the kidneys of mice after 45 min of ischemia followed by reperfusion for 6–48 h. Renal IRI induced a reperfusion time dependent increase in NETs, reaching a peak approximately 24 h after reperfusion and decreasing thereafter. At 24 h, immunofluorescence revealed amorphous extracellular DNA structures colocalized with CitH3 and Ly6G in tubules. Protein levels of CitH3 were barely detectable in the kidneys of control and sham surgery mice but dramatically increased 24 h after renal IRI (**Figures 2B, C**).

### Kidney Injury was Alleviated After Neutrophils Depletion

**Figure 3:**
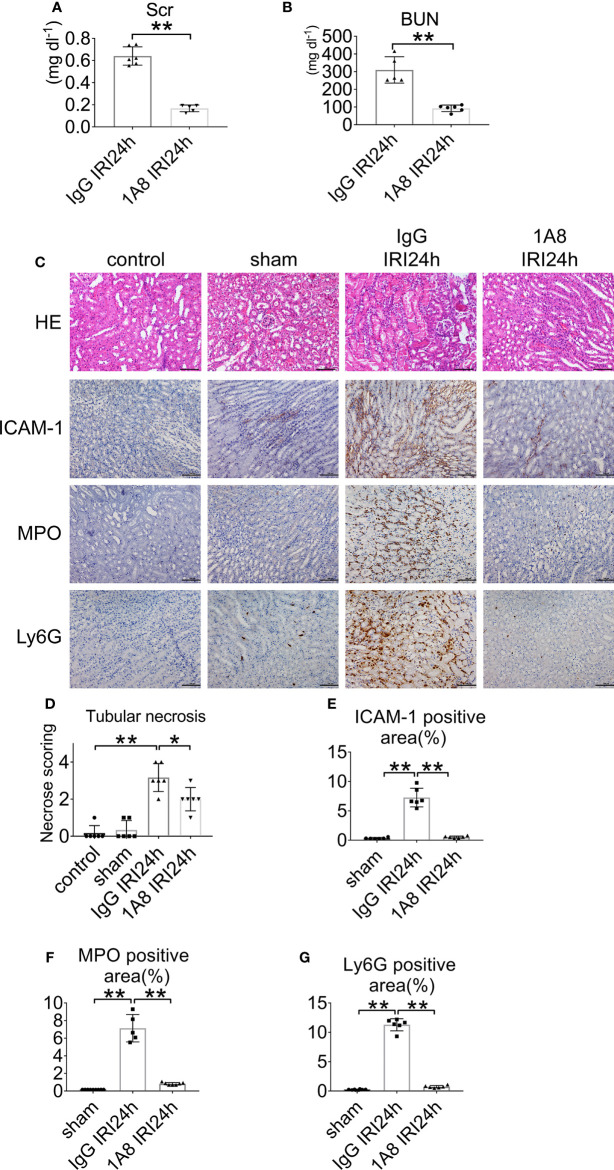
Neutrophil depletion preserves renal function in IRI mice. **(A)** Serum creatinine, and **(B)** urea nitrogen of WT mice injected with 1A8 or IgG, n = 5–6/group. **(C)** Histology of IRI kidney in the indicated treatments. HE staining (Row 1). ICAM-1 immunohistochemistry (Row 2). MPO immunohistochemistry (Row 3). Ly6G immunohistochemistry (Row 4). Scale bar = 200 μM. n = 5–6/group. The percentage of positive neutrophil staining in 10 non-overlapping fields was quantified using Image J software. **(D)** Score for histopathology of tubular damage after renal IRI using HE-stained renal tissue sections. **(E)** ICAM-1-positive area. **(F)** MPO-positive area. **(G)** Ly6G-positive area. *P < 0.05, **P < 0.01. Data are presented as mean ± SD.

To assess the role of neutrophils in the development of AKI, mice were depleted of neutrophils *via* anti-Ly6G (1A8) injection at 24 and 2 h prior to IRI. Control groups received an equivalent concentration of IgG. The neutrophil number decreased by >90% in the blood of mice treated with mAb 1A8 compared with in IgG-treated control mice 24 h after IRI (**Supplementary Figure S2A**), and neutropenia was observed in renal sections (**Supplementary Figures S2B, C**). IgG-injected mice had elevated levels of serum creatinine and urea. In contrast, mice with neutrophils depletion had lower levels of indices for renal function (**Figures 3A, B**). In mice pretreated with 1A8, there was little necrosis or cast formation in the cortex compared with mice pretreated with IgG (**Figures 3C, D**). There was decreased neutrophils infiltration in the 1A8 group compared with the IgG treated group (**Figures 3E–G**).

### ETs are Mainly Derived From Neutrophils in IRI and NETs Clearance Reduces Renal Injury

**Figure 4:**
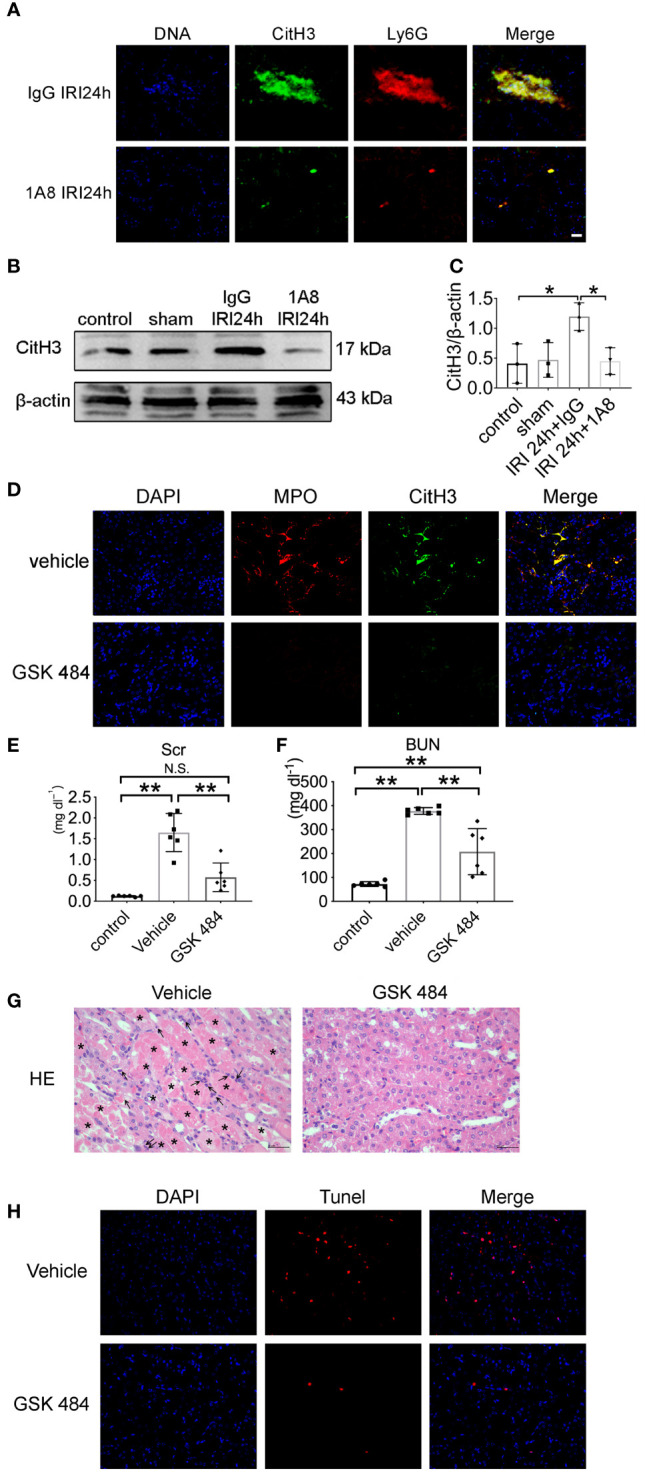
NET formation decreased in renal tissue after neutrophils depletion. **(A)** Representative overlay images of NETs staining after neutrophil depletion by injection of 1A8 and control IgG mice 24 h after IRI. DAPI (blue), CitH3 (green), Ly6G (red). **(B)** Immunoblotting analysis of CitH3 expression in mouse kidneys 24 h after IRI. **(C)** Quantitative analysis was analyzed by normalization to β-actin expression, n = 3/group. **(D)** Representative NETs staining of bilateral IRI at different time points after reperfusion. Colocalization of CitH3 (green), MPO (red), and swelled nuclei (blue). **(E)** Serum creatinine, and **(F)** urea nitrogen of IRI mice injected with vehicle or GSK 484, n = 6/group. **(G)** Representative HE section of the kidney from mice in each group 24 h after IRI. Arrows denote tubules with necrosis and detachment. Asterisks indicate neutrophils infiltration. **(H)** Representative TUNEL staining images in kidney. *P < 0.05, **P < 0.01. N.S., No Significance. Data are presented as mean ± SD.

To determine whether infiltrating neutrophils constitute ETs after IRI injury, we further examined the effect of 1A8-mediated neutrophil depletion on the expression of NETs. In the IgG group, postischemic kidneys displayed CitH3- and Ly6G-positive areas. The 1A8 group revealed a more significant reduction in NETs formation 24 h after reperfusion, indicating that neutrophils depletion decreased NETs formation (**Figure 4A**). The expression of CitH3 was higher in the control group than in the 1A8 group (**Figures 4B, C**). These data suggest that ETs released from neutrophils contribute to renal necroinflammation in AKI.

To verify the role of NETs in IRI, we used PAD4 inhibitors. The PAD4-specific inhibitor GSK 484 was found to reduce the release of NETs (**Figure 4D**). Compared to vehicle, GSK 484 significantly improved renal function (**Figures 4E, F**). HE staining of kidney from vehicle treated IRI mice exhibits severe renal tubular damage, as indicated by tubular necrosis, loss of brush boundary, and cast formation. Notably, GSK 484 therapy reduces these structural damages (**Figure 4G**). TUNEL fluorescence staining in IRI mice reveals that inhibiting PAD4 reduces apoptosis (**Figure 4H**).

### Lack of C3 Ameliorates AKI

**Figure 5:**
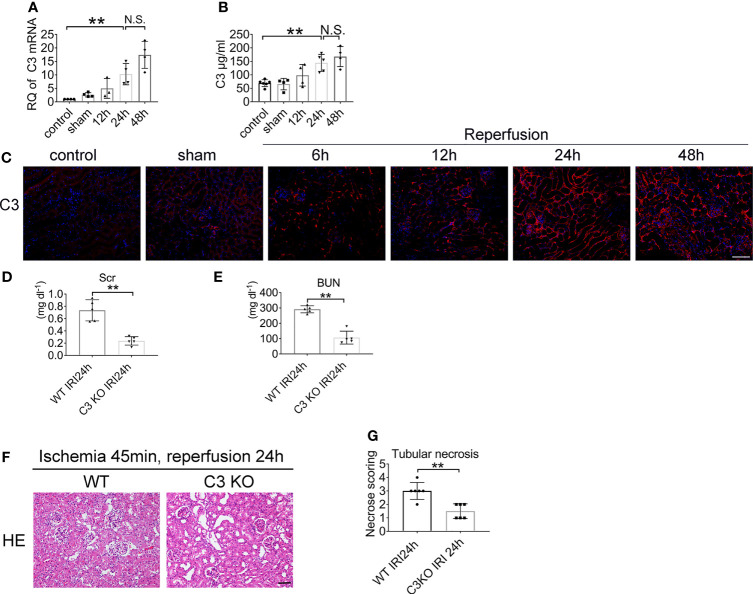
Renal IRI leads to C3 activation and C3 deficiency ameliorates IRI. Levels of C3 in renal tissue after IRI or sham surgery in WT mice were measured by qPCR **(A)** and ELISA **(B)**, n = 4–6/group. **(C)** Representative immunofluorescence microscopy image of C3 staining in kidney from WT mice subjected to renal IRI, n = 6/group. Plasma creatinine **(D)**, and urea nitrogen **(E)** 24 h after IRI in WT and C3 KO mice, n = 5–6/group. **(F)** Representative HE staining of kidney 24 h after IRI in WT and C3 KO mice. n = 5–6/group. **(G)** Score for histopathology of tubular damage after renal IRI using HE stained renal tissue sections. **P < 0.01. N.S., No Significance. Data are presented as mean ± SD.

To determine the biological role of C3 in renal IRI, we first assessed the expression and distribution of C3. The analysis demonstrated increased C3 mRNA expression in the kidney after renal ischemia and 12, 24, and 48 h of reperfusion. Low C3 mRNA levels were detected after 12 h of reperfusion and were markedly increased in the kidney 24 h after IRI. The mouse GAPDH probe was used to demonstrate equal loading of lanes (**Figure 5A**). Moreover, ELISA confirmed that the level of C3 also tended to increase over time (**Figure 5B**). The expression of C3 in the 48 h group looked different than that in the 24 h group, but the difference was not statistically significant. Overall, renal IRI resulted in a reperfusion time-dependent increase in C3 in renal tissue (**Figure 5C**).

To determine whether C3 is involved in renal IRI, we assessed serum creatinine and serum urea levels. Renal functions were improved in C3 KO mice after 24 h of reperfusion compared to the WT mice (**Figures 5D, E**). HE staining showed more severe tubular injury in the outer medulla in WT mice than in C3 KO mice (**Figures 5F, G**). These data demonstrated that C3 plays a role in renal IRI.

### Neutrophil Clearance Does not Affect C3 Expression Levels

**Figure 6:**
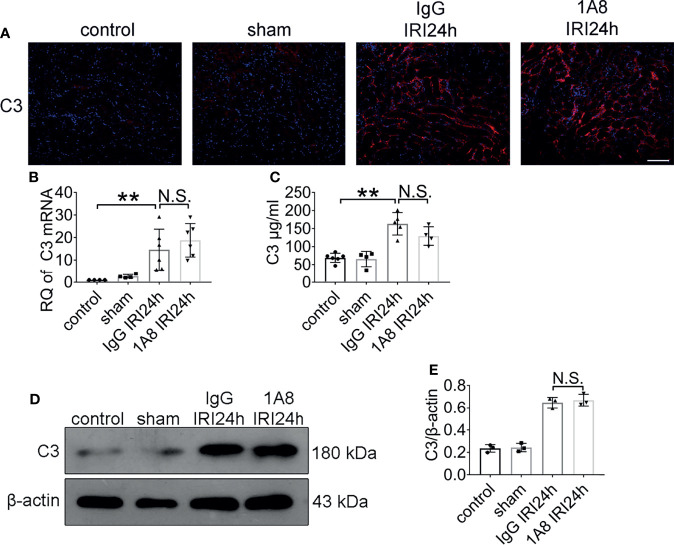
The expression of C3 did not change after neutrophil depletion. Levels of C3 in sham-operated mice and bilateral IRI kidneys in 1A8 and IgG mice (ischemia for 45 min and reperfusion for 24 h) were measured by IF **(A)**, qPCR **(B)**, and ELISA **(C)**, n = 4–6/group. **(D)** Immunoblotting analysis of C3 (expected weight: 180 kDa) expression in 1A8 and IgG mouse kidneys 24 h after IRI. **(E)** Quantitative analysis was analyzed by normalization to β-actin expression, n = 3/group. **P < 0.01. N.S., No Significance. Data are presented as mean ± SD.

To identify whether neutrophils depletion affects C3 level, mice with bilateral renal IRI (ischemia for 45 min and reperfusion for 24 h) underwent treatment with 1A8 to deplete neutrophils, or control IgG. Immunofluorescent staining results indicated that the level of C3 does not only depend on neutrophils presence (**Figure 6A**). Compared to sham mice, C3 mRNA levels were higher in IgG and 1A8 mice undergoing IRI surgery (**Figure 6B**). In addition, no difference in the protein level of C3 in the kidney was observed (**Figures 6C–E**). These results indicated that neutrophils cannot trigger the C3 secretion increase directly.

### C3 is Essential for Neutrophils Infiltration and NETs Formation After IRI

**Figure 7:**
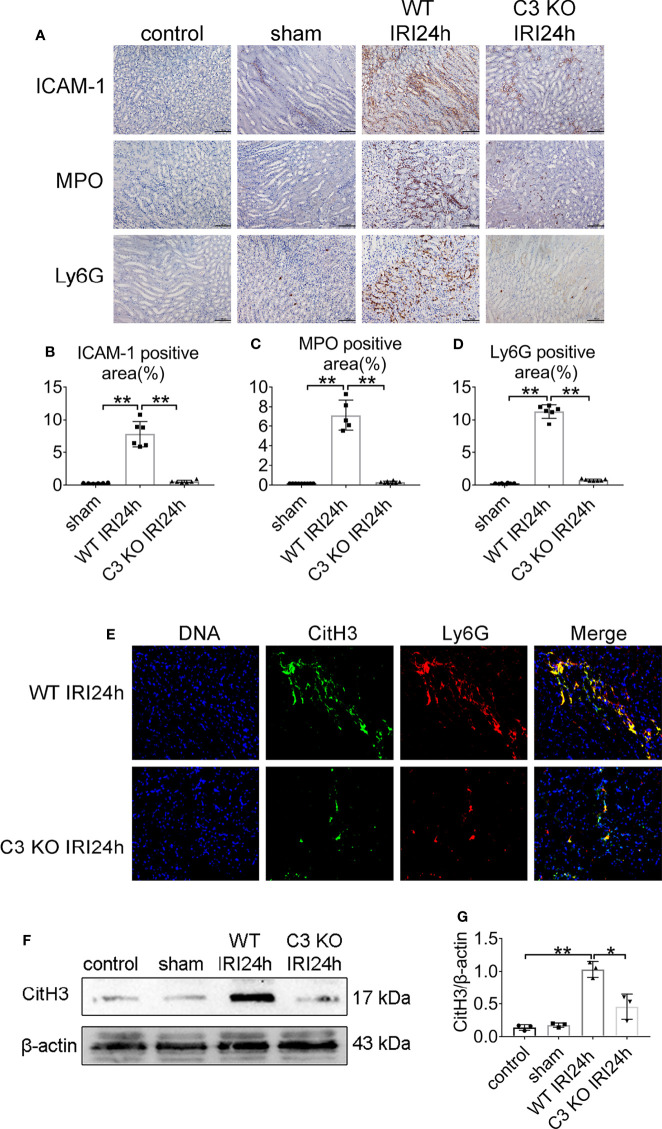
NET formation decreased in C3 deficient mice. **(A)** Histology of IRI kidney in the indicated treatments. ICAM-1 immunohistochemistry (Row 1). MPO immunohistochemistry (Row 2). Ly6G immunohistochemistry (Row 3). Scale bar = 100 μM, n = 5–6/group. The percentage of positive neutrophil staining in 10 non-overlapping fields was quantified using Image J software. **(B)** ICAM-1-positive area. **(C)** MPO-positive area. **(D)** Ly6G-positive area of sections from kidneys harvested for 24 h in WT and C3 KO mice. **(E)** Representative overlay images of NETs staining in WT and C3 KO mice 24 h after IRI, DAPI (blue), CitH3 (green), and Ly6G (red). **(F)** Immunoblotting analysis of CitH3 expression in WT and C3 KO mouse kidneys 24 h after IRI. **(G)** Quantitative analysis was analyzed by normalization to β-actin expression.*P <0.05, **P < 0.01.

To examine whether C3 contributes to triggering neutrophils activation, we assessed the level of neutrophils and NETs in male WT and C3 KO mice at 24 h after reperfusion. As shown in **Figures 7A–D**, neutrophils were hardly detectable in the sham mice. IRI induced significant neutrophils infiltration in WT mice, and to a lesser extent, in C3 KO mice 24 h after IRI. Next, we tested whether C3 deficiency reduced the expression of NETs. Immunofluorescence microscopy showed that Ly6G and CitH3 were dramatically increased in the WT mice 24 h after IRI but were barely detectable in the C3 KO mice (**Figure 7E**). Moreover, the protein expression of CitH3 in WT mice was higher than in C3 KO mice (**Figures 7F, G**). These results showed that C3 could induce NETs.

### C3a is a Crucial Molecule in IRI

**Figure 8:**
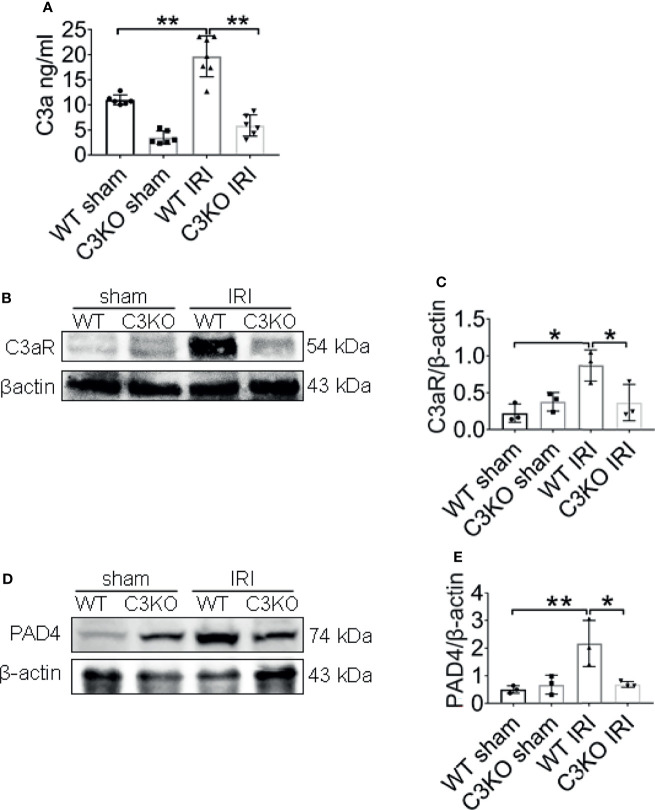
C3a is an important molecule in IRI. **(A)** Renal levels of C3a from WT and C3KO mice, measured by ELISA. **(B)** Immunoblotting analysis of C3aR expression in mouse kidneys 24 h after IRI. **(C)** Quantitative analysis was analyzed by normalization to β-actin expression, n = 3/group. **(D)** Immunoblotting analysis of PAD4 expression in mouse kidneys 24 h after IRI. **(E)** Quantitative analysis was analyzed by normalization to β-actin expression, n = 3/group. *P < 0.05, **P < 0.01. Data are presented as mean ± SD.

To further explore the underlying molecular mechanisms involved in C3 and NETs induction, the expression of C3a, C3aR, and PAD4 were examined in ischemia renal. C3a protein levels in the renal tissue increased in WT IRI mice compared with those in the sham group (**Figure 8A**). At the same time, the protein levels of C3aR, the receptor of complement C3a, were significantly upregulated after IRI surgery (**Figures 8B, C**). The C3 KO markedly limited the progressive rise ofC3a and C3aR in kidneys of IRI mice. Western blotting analysis showed that PAD4 expression was significantly increased in WT IRI mice. In contrast, there was no significant change between the sham group and the C3 KO group (**Figures 8D, E**).

### C3a Primed Neutrophils to Form NETs

**Figure 9:**
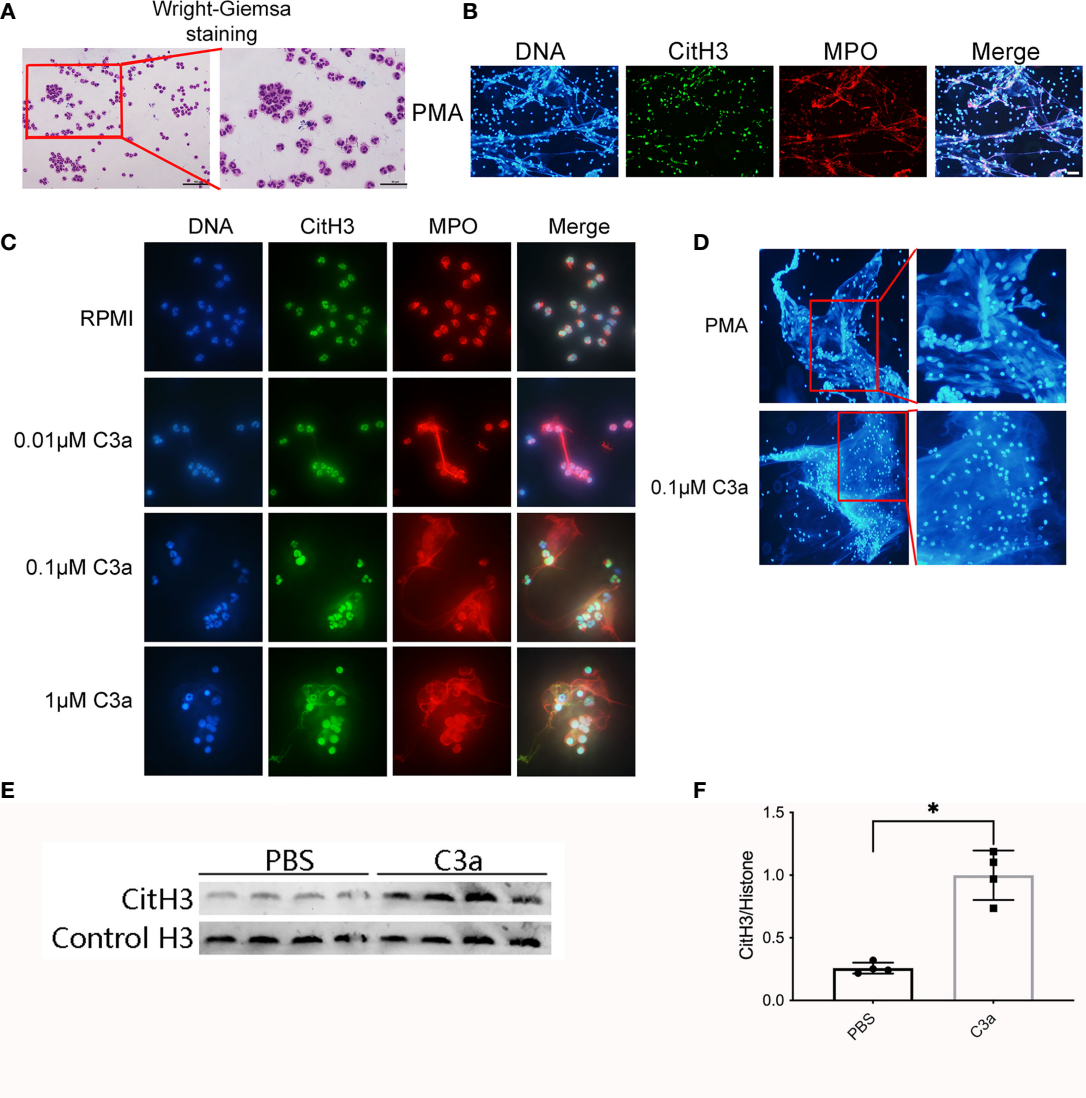
Neutrophils release NETs in response to PMA and C3a. **(A)** Wright–Giemsa staining of neutrophils isolated from peripheral blood of normal individuals. Representative overlay images of NETs from human granulocytes treated with 15 nM PMA **(B)**, and C3a of different concentration **(C)**. DAPI (blue), CitH3 (green), and MPO (red). **(D)** The contrast diagram of “suicidal” and “vital” NETosis induced by PMA and C3a. **(E)** Immunoblotting analysis of CitH3 expression in neutrophil stimulated by C3a. **(F)** Quantitative analysis was analyzed by normalization to β-actin expression. *P < 0.05 versus PBS.

To verify that incubation of C3 with granulocytes results in the formation of NETs, we isolated neutrophils from healthy volunteers. The purity of neutrophils was >95%, as determined by Wright–Giemsa staining (**Figure 9A**). Neutrophils isolated from human peripheral whole blood were cultured at 37 ℃ for 4 h with PMA, RPMI, different concentrations of C3a. Neutrophils treated with PMA for 4 h, lost their morphology and released NETs, characterized by the loss of the lobate nucleus morphology, chromatin decondensation, and mixture of DNA and granular proteins (MPO) (**Figure 9B**). Approximately 0.1 μM C3a stimulated NETs remaining intact neutrophils phenotype with lobulated nuclei while 1 μM C3a stimulating NETs with loss of lobate nucleus morphology (**Figure 9C**). It was found that PMA-induced NETs nuclear decondensation, while 0.1 μM C3a-induced NETs nucleus still showed obvious lobularucleus. Due to chromatin decondensed nuclear diffusion, the chromatin volume of PMA-induced NETs was larger than that induced by C3a (**Figure 9D**). Western blot analysis of C3a-stimulated neutrophils revealed an enhancement of CitH3 (**Figures 9E, F**). In conclusion, our data reveal a complex interplay between C3a and neutrophils, which can induce both “suicidal” and “vital” NETosis, depending on the concentration of C3a.

## Discussion

The incidence of AKI increase and its influence on long-term health and cost is far greater than previously acknowledged (20). AKI can be caused by a broad range of etiologies, and IRI is one of the most common causes. Although the formation of ETs in the kidney after IRI has been demonstrated to be crucial in the pathogenesis of AKI, the origin of ETs and the relationship between C3 and ETs have not been investigated to date. Our results suggest that following renal IRI, there was an increase in infiltration of neutrophils, formation of NETs, and expression of C3 within the kidney, which correlated with renal injury. Inhibiting NET formation by neutrophils depletion using 1A8 or C3 KO significantly ameliorated the resultant kidney condition, while neutrophils did not affect the expression of C3.

Representative anti-MPO and anti-Ly6G staining showed increased neutrophils infiltration in the renal corticomedulla in the IRI group compared to the sham group. The corticomedullary regions are susceptible to ischemia reperfusion because of high oxygen demand (21). Our findings suggest that in bilateral renal IRI clamped for 45 min, kidney interstitial neutrophils content increased significantly, peaking at 24 h. Once recruited to the injury site, they initiate a multistep inflammatory response (22). Neutrophils mobilization to blood, recruitment to the kidney, and plasm creatinine and urea were lower in neutrophils-depleted mice. Thus, neutrophils mediate renal IRI-induced injury. Suppression of neutrophils recruitment is likely to limit acute injury to the kidney, particularly the following reperfusion.

Recent studies have indicated that not only neutrophils but also other immune cells, such as macrophages, can form traps (6). Double staining for CitH3 and granulocytes in the corticomedullary region confirmed that renal NETs were granulocyte-specific NETs. To further study the effect of neutrophils depletion on NET formation, we performed NETs staining of kidney sections of mice treated with control IgG or 1A8 and subjected them to renal IRI. We found that depleting neutrophils during IRI abrogated intrarenal NETs release and further improved renal function. These results indicate that the formation of NETs released by neutrophils may be responsible for renal injury. Neutrophils depletion *in vivo* models has been achieved by cytotoxic chemotherapy agents, such as vinblastine or cyclophosphamide. However, it causes myelosuppression and also nonspecific suppress other leukocytes (23, 24). Therefore, antibody-mediated depletion is more specific for depleting neutrophils (25). The most commonly used antibody is mAb RB6-85C. Although RB6-5Cb targets the Ly6G antigen on neutrophils, it also binds to the Ly6C antigen, which is expressed on dendritic cells, monocytes, macrophages, and lymphocytes (26, 27). To circumvent this issue, we instead used a novel anti-Ly6G-specific mAb, 1A8. 1A8 was demonstrated to selectively deplete neutrophils and preserve other cell types (23, 28).

The importance of NETs in AKI has increasingly been appreciated. Programmed ‘disarming’ of the neutrophil resulted in a blunted capacity to produce NETs, rendering the neutrophils less toxic before they reached tissues (29). A growing number of researches have recently revealed that degradation NETs components such histone and DNA could be used as the therapeutic strategy to attenuate I/R injury in the kidney. PAD4-deficient mice did not form NETs and were partially protected from renal ischemia/reperfusion-induced AKI (30). DNase-1 treatment attenuates histopathological changes after intestinal I/R injury. Administration of recombinant thrombomodulin may have a protective impact on the lungs after renal I/R by blocking histone and NET accumulation (31). We found that renal injury after IRI was accompanied by an increase in PAD4 and that GSK 484, a PAD4-specific inhibitor, reduced renal injury, suggesting that PAD4 mediates the release of NETs during IRI and that NETs can indeed directly damage the renal tubules. Collectively, these studies mainly focus on NETs degradation. If the formation of NETs can be inhibited upstream, the toxic effects of NETs may be better alleviated. Given the central role of complement in IRI, we hypothesized that complement is a crucial upstream mechanism driving NET formation in AKI.

The lectin pathways, alternative and classical pathways, are involved in IRI, and all of these pathways converge on C3 (32). Complement C3 is abundantly present in plasma following ischemia reperfusion, and its access to the interstitial space is increased (15). In our research, we found that the expression of C3 at each temporal point after IRI increased significantly with concomitant renal dysfunction, while C3 KO mice were protected. Accumulated evidences indicate that C3-targeted intervention has clinical promise as a viable therapeutic similar to anti-C5 therapeutics (33).

The interplay of the complement system and NETs has been demonstrated in many studies (19, 34). A recent study uncovers that targeting complement activation or/and NET formation can disrupt the vicious cycle of COVID-19 (35). Our study found that IRI-induced neutrophils infiltration and NETs release were suppressed in C3 KO. In agreement with our proposal, it has been demonstrated that C3 KO mouse neutrophils cannot release histones or nuclear DNA to make NETs during acute gram-positive bacterial infections (36). However, our research did not reveal significant differences in the expression of C3 between 1A8-injected and IgG-injected mice, indicating that neutrophils or NETs cannot directly induce C3 expression in IRI. In contrast, others showed that NETs could activate complement in SLE and AAV (37, 38). These contrasting results may be due to several reasons. First, the disease model in each of these studies was different and not comparable. Second, neutrophils are not the only cells that can produce C3. IRI results in rapid and massive complement activation by resident innate immune cells and parenchymal cells. Studies have demonstrated that locally synthesized C3 seems to have a more substantial influence than circulating C3 (39, 40). IRI can stimulate tubular epithelium cells to produce C3 (41–43). Endothelial and mesangial cells can also induce local complement (44). Furthermore, resident kidney macrophages can secrete C3 (39). From all these studies, it is apparent that the relationship between neutrophils and C3 in IRI has remained a conundrum until now. As inflammatory cells in the early stages of IRI, neutrophils are likely to be involved in C3 secretion as well. Complement C3 activation was accompanied by the generation of C3a, the active complement fragment. C3aR is highly expressed on neutrophils’ surfaces, and C3a can activate neutrophils by binding to receptors on their surfaces. Activated neutrophils then release NETs, exacerbating tissue damage. Our *in vivo* and *in vitro* research can back this up. Additionally, increased PAD4 expression was significantly associated with high thrombin activity caused by citrullinated antithrombin (45).

The rationale for this study is that during IRI, C3 synthesis by liver-derived circulating and also renal parenchymal and inflammatory cells increases significantly. C3 activation can boost neutrophil infiltration and induce inflammation. We used 1A8 to clear neutrophils from mice and discovered a decrease in extracellular traps (ETs), indicating that neutrophils are the primary source of ETs. During IRI, GSK 484 inhibited the creation of NETs, which reduced renal tissue injury, implying that NETs can increase tissue injury. In C3 KO mice, neutrophil infiltration was dramatically reduced, as was NETs release, implying that C3 may be the source of NETs released from neutrophils. *In vitro*, C3a can induce neutrophils to release NETs. They were further suggesting that C3 may be involved in the release of NETs from neutrophils.

**Figure 10:**
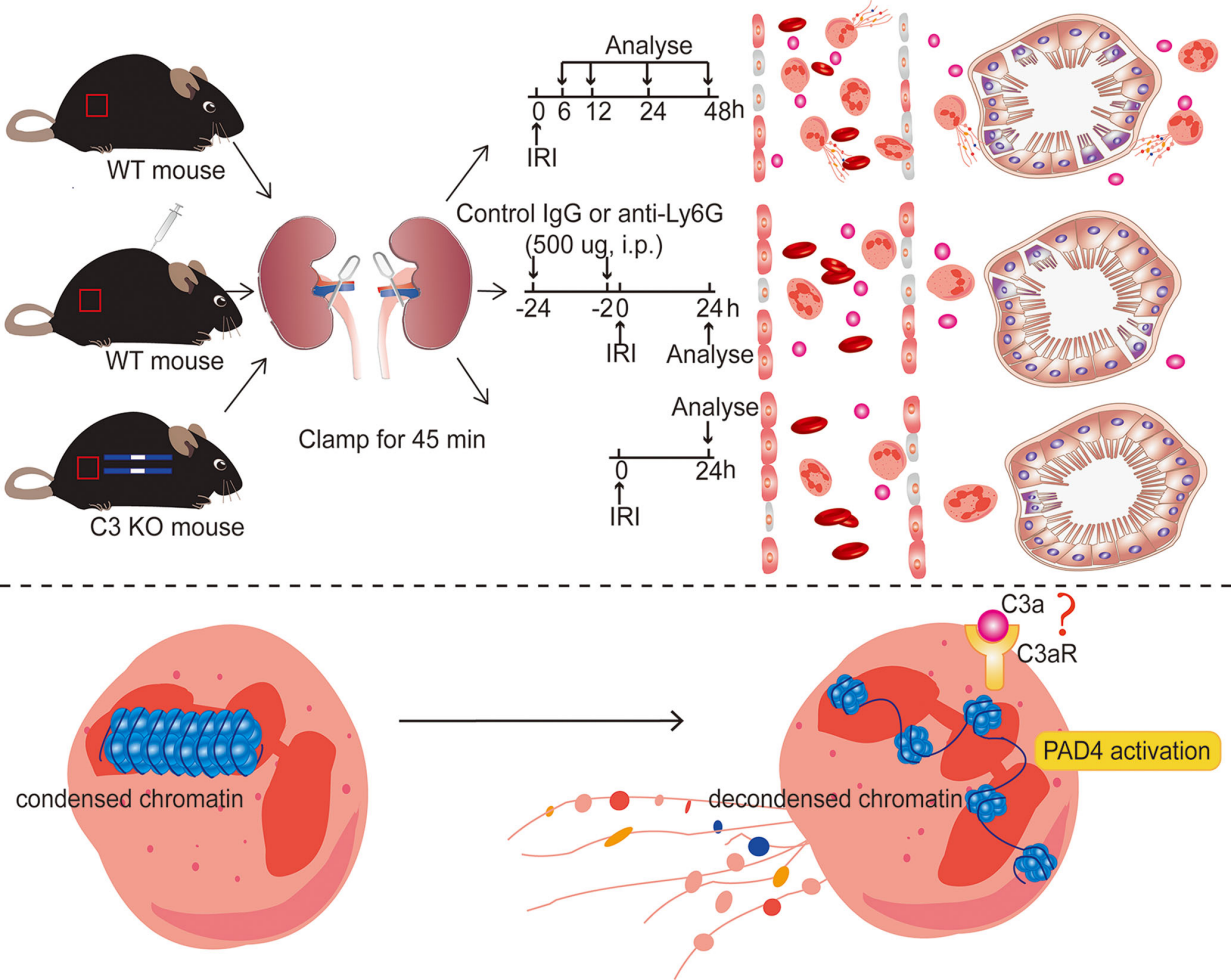
Diagram of the underlying mechanism in renal IRI-induced AKI. Inhibiting NET formation by neutrophils depletion using anti-Ly6G (1A8) or C3 KO significantly ameliorated the resultant renal injury. In ischemic AKI, there is increased cleavage of C3 to C3a, which probably binds to C3aR on the surface of neutrophils and then activates PAD4, leading to the release of NETs.

There were some limitations in our study. We evaluated only the effect of pretreatment with 1A8 in IRI prevention.It remains unclear whether 1A8 will have a similar impact when administered after the onset of the injury. Although our research demonstrated that neutrophils infiltration, NET formation, and C3 increase are essential mediators of renal IRI, the mechanism by which C3 triggers neutrophils release of NETs after IRI and the mechanism by which NETs induce tissue injury remain unknown and require further study. Based on *in vivo* and *in vitro* findings, we hypothesize C3a binds to C3aR on the surface of neutrophils, activates neutrophils, and promotes NETs with the assistance of PAD4 (**Figure 10**). This assumption should be explored further. Our results are also limited by the differences in the immune systems between mice and humans. For example, we found that neutrophil levels in mice and humans were not very consistent, with mice having a lower proportion of peripheral blood neutrophils. Despite these limitations, our research still provides some new viewpoints for the future treatment of IRI.

Our results suggest that infiltration of complement C3 leads to neutrophils activation and then results in NET formation and subsequent renal damage.

## Data Availability Statement

The original contributions presented in the study are included in the article/**Supplementary Material**. Further inquiries can be directed to the corresponding author.

## Ethics Statement

All animal protocols were reviewed and approved by the Institutional Animal Care and Use Committee of Fujian Medical University (Approval Number: 2017-062).

## Author Contributions

XW and JW designed the study. XW, DY, LL, and CX performed experiments. JC, YC, LY, and GL analyzed data. XW wrote manuscript. JW revised the manuscript. All authors listed have made a substantial, direct, and intellectual contribution to the work and approved it for publication.

## Funding

This work was supported by the Joint Funds for the Innovation of Science and Technology of Fujian province (Grant number: 2019Y9117), the Startup Fund for Scientific Research of Fujian Medical University (Grant number: 2019QH2035).

## Conflict of Interest

The authors declare that the research was conducted in the absence of any commercial or financial relationships that could be construed as a potential conflict of interest.

## Publisher’s Note

All claims expressed in this article are solely those of the authors and do not necessarily represent those of their affiliated organizations, or those of the publisher, the editors and the reviewers. Any product that may be evaluated in this article, or claim that may be made by its manufacturer, is not guaranteed or endorsed by the publisher.
