# Supplementary material for: Reduced Neutrophil Extracellular Trap Formation During Ischemia Reperfusion Injury in C3 KO Mice: C3 Requirement for NETs Release
Source: Front Immunol. 2022 Feb 16;13:781273. doi: 10.3389/fimmu.2022.781273 (PMC8889019; doi:10.3389/fimmu.2022.781273)
Supplement: Supplementary file 1 [file DataSheet_1.docx]

Supplementary Material

**Supplementary Figure 1**

**
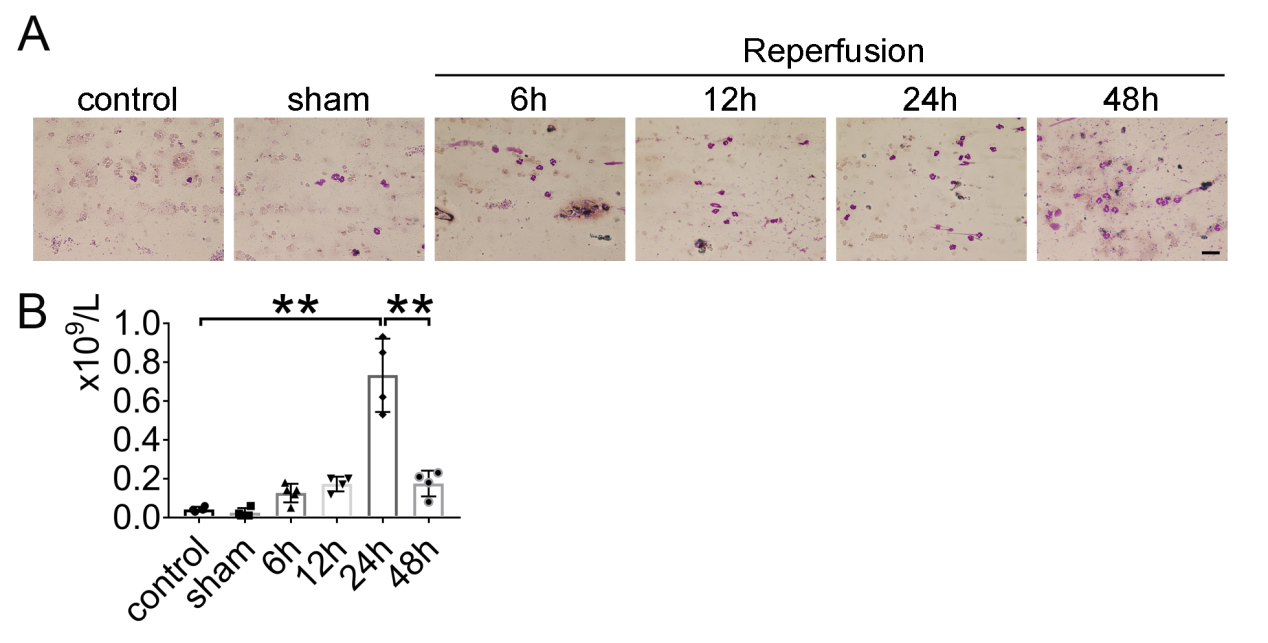
**

**Suppl. Figure 1. Change in neutrophils.** (A) Morphological characteristics of neutrophils by Wright-Giemsa staining, n=6/group. (B) Peripheral blood neutrophil count, n=4-5/group. *P<0.05 versus respective control, **P<0.01 versus respective control. Data are presented as mean ± SD.

**Supplementary Figure 2**

**
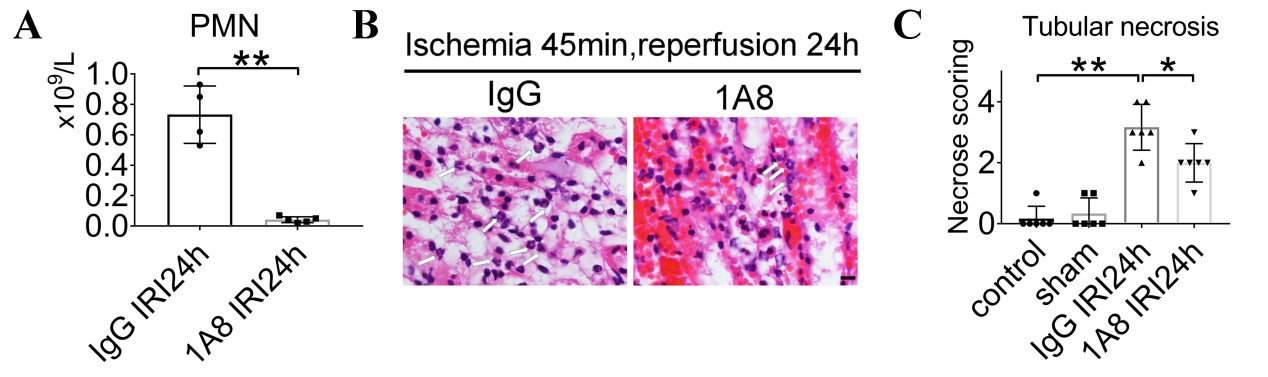
**

**Suppl. Figure 2.** Neutrophils depletion by injection of anti-Ly6G (1A8) 24 and 2 h before the surgery. (A) Effect of 1A8 on peripheral blood neutrophil counts, n=4-5/group. (B) HE staining of kidney sections from 1A8 and IgG mice 24 h after IRI. White arrows indicate infiltrating neutrophils, n=6/group. (C) Quantification of lesion neutrophils in different treatments, n=4-5/group. *P<0.05 versus respective control, **P<0.01 versus respective control. Data are presented as mean ± SD.

**Full unedited gel**

**
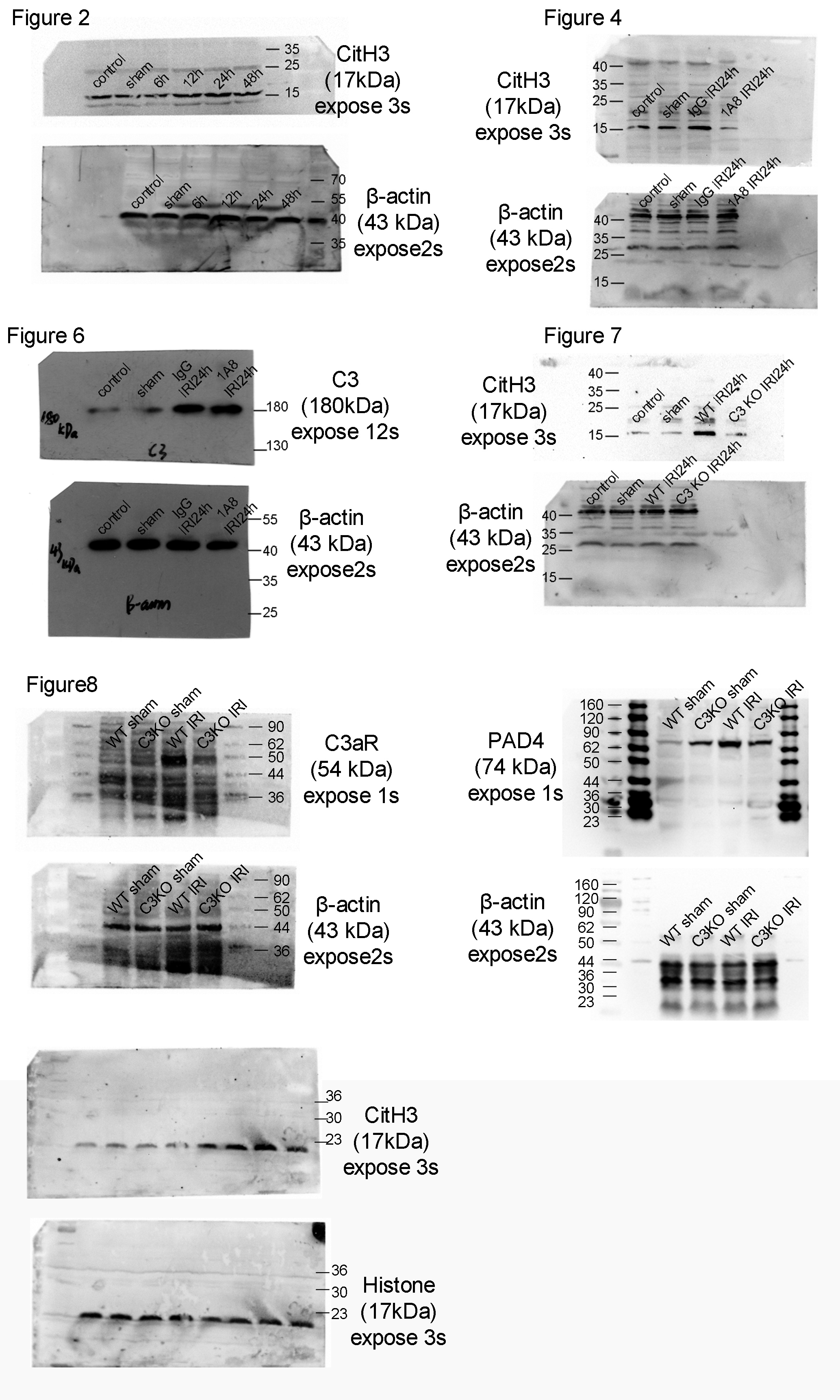
**
